# Th17 Immunity in Children with Allergic Asthma and Rhinitis: A Pharmacological Approach

**DOI:** 10.1371/journal.pone.0058892

**Published:** 2013-04-03

**Authors:** Giusy Daniela Albano, Caterina Di Sano, Anna Bonanno, Loredana Riccobono, Rosalia Gagliardo, Pascal Chanez, Mark Gjomarkaj, Angela Marina Montalbano, Giulia Anzalone, Stefania La Grutta, Fabio Luigi Massimo Ricciardolo, Mirella Profita

**Affiliations:** 1 Unit: “ Ex vivo/In vitro models to study the Immunopathology and the Pharmacology of airway diseases”, Institute of Biomedicine and Molecular Immunology, Italian National Research Council, Palermo, Italy; 2 Dipartimento Biomedico di Medicina, Interna e Specialistica, Sezione di Pneumologia, University of Palermo, Palermo, Italy; 3 Département des Maladies Respiratoires, AP-HM, Université de la Méditerranée, Marseille, France; 4 INSERM-CNRS U600, UMR6212, Université de la Méditerranée, Marseille, France; 5 Environmental Health Unit, ARPA Palermo, Palermo, Italy; 6 Division of Respiratory Disease, Department of Clinical and Biological Sciences, University of Torino, Torino, Italy; University of Oslo, Norway

## Abstract

Th17 cells and IL-17A play a role in the development and progression of allergic diseases. We analyzed the IL-17A levels in sputum supernatants (Ss), nasal wash (NW) and plasma (P) from Healthy Controls (HC) and children with Asthma/Rhinitis. We tested the expression of IL-17A, RORγ(t) and FOXP3 in peripheral blood T-lymphocytes from intermittent and mild-moderate asthma. The effect of Budesonide and Formoterol was tested “*in vitro*” on IL-17A, RORγ(t) and FOXP3 expression in cultured T-lymphocytes from mild-moderate asthma/persistent rhinitis patients, and on nasal and bronchial epithelial cells stimulated with NW and Ss from mild-moderate asthma/persistent rhinitis. Further, the effect of 12 weeks of treatment with Budesonide and Formoterol was tested “*in vivo*” in T-lymphocytes from mild-moderate asthma/persistent rhinitis patients. IL-17A was increased in Ss, NW and P from children with mild-moderate asthma compared with intermittent and HC. In cultured T-lymphocytes IL-17A and RORγ(t) expression were higher in mild-moderate asthma/persistent rhinitis than in mild-moderate asthma/intermittent rhinitis, while FOXP3 was reduced. Budesonide with Formoterol reduced IL-17A and RORγ(t), while increased FOXP3 in cultured T-lymphocytes from mild-moderate asthma/persistent rhinitis, and reduced the IL-8 release mediated by IL-17A present in NW and Ss from mild-moderate asthma/persistent rhinitis in nasal and bronchial epithelial cells. Finally, Budesonide with Formoterol reduced IL-17A levels in P and Ss, CD4^+^IL-17A^+^T-cells, in naïve children with mild-moderate asthma/persistent rhinitis after 12 weeks of treatment. Th17 mediated immunity may be involved in the airway disease of children with allergic asthma and allergic rhinitis. Budesonide with Formoterol might be a useful tool for its therapeutic control.

## Introduction

Allergic diseases, including rhinitis (AR) and asthma, are chronic inflammatory disorders with a prevailing Th2 immune response. The inhalation of allergens leads to hyperreactivity, recruitment of eosinophils, mast cells, and lymphocytes in the upper and lower airways triggering the inflammatory cascade and generating local and systemic inflammatory responses. The presence of an uncontrolled inflammation in the upper and lower airways as well as in the systemic circulation may compromise the control of AR and asthma with a consequent progression of the diseases [1].

T helper (Th) cells, both Th1 and Th2 cells, play an important role in the initiation and challenge phases of AR and asthma [2], [3]. Recent studies have identified an IL-23-dependent T-cell population, a Th-cell lineage distinct from Th1 and Th2 called Th17, which plays an important role in inflammation and tissue injury. Th17 cells primarily produce Interleukin-17A (IL-17A) by CD4 and CD8 T-cells of both Th1 and Th2 cytokine profiles that differentiate in Th17 via the activation of the nuclear receptor retinoic acid–related orphan receptor γt (RORγt). IL-17A is overexpressed in the lung during acute neutrophilic inflammation and asthma [4]. A number of recent studies have examined the effect of IL-17A on IL-8 secretion by airway epithelial cells and airway smooth muscle cells [5], [6]. Furthermore, it was observed that IL-17A induces epigenetic changes which in turn diminish the ability of glucocorticosteroids (GC) to inhibit IL-8 production from human bronchial epithelial cells [7].

A balance between Th17 and regulatory T-cells (Tregs) is crucial for immune homeostasis. Both an excess in Th17 function, or increased numbers of Th17, and a defect in Treg function or reduced numbers of Treg, may trigger the development and progression of inflammatory diseases, including allergic asthma and rhinitis. Tregs dominantly express Forkhead family transcription factor Foxp3 which activates many suppressive genes in Tregs and inhibits many effector T cell genes. Pharmacological treatment might restore the balance between Th17 and Treg promoting the resolution of inflammation in airway diseases [8]. In this context, the impact of current pharmacological interventions is worth to be investigated to counteract the mechanism of crosstalk between innate and adaptive immunity associated with Th17 immunity and IL-17A activity during the allergic process.

To better understand the role of IL-17A in asthma and rhinitis we investigated: 1) the levels of IL-17A in sputum supernatants (Ss), in nasal wash (NW) and in plasma (P), and 2) the levels of intracellular IL-17A, RORγ(t) and FoxP3 expression in peripheral T-lymphocytes. Furthermore, we performed *in vitro* experiments to test whether the IL-17A present in induced sputum (Ss) and in NW may compromise the release of IL-8 from bronchial and nasal airway epithelial cells, and whether the intracellular IL-17A expressed in T-cells from children with asthma and AR is involved in the Th17/Treg balance. Finally, we tested the effect of Inhaled Corticosteroids (ICS) and long-actingβ2agonists (LABA) in both an in vitro and in vivo approach in order to provide new therapeutic strategies to control the inflammation associated with Th17 producing IL-17A in children with AR and concomitant asthma.

## Materials and Methods

### Subjects

Pediatric subjects (age between 8 and 17 years) were recruited among outpatients attending the Pulmonology/Allergy Clinic of the Italian National Research Council in Palermo. Asthma diagnosis and assessment of severity were performed according to Global Initiative for Asthma (GINA) guidelines [9]. AR diagnosis was performed at the study entry according to Allergic Rhinitis and its Impact on Asthma (ARIA) guidelines [10]. The patients were divided in two groups: 15 children had intermittent asthma (IA) (treated with short-acting β2-agonists on demand during the previous 3 months) and 19 had mild to moderate asthma (MA). Eight IA patients and 9 MA patients had concomitant intermittent allergic rhinitis (IR); 7 IA patients and 10 MA patients had concomitant persistent allergic rhinitis (PR). The control group was composed of 16 healthy children (HC), tested for allergy to exclude the allergic disease. No patients had nasal polyposis or bronchial or respiratory tract infections or had a severe exacerbation of asthma resulting in hospitalization during the last month. Within 2 days from the collection of Ss, NW, and blood samples, all subjects performed pulmonary function tests as recommended by the GINA guidelines [9].

To assess *in vivo* the effect of the treatment with inhaled GC and LABA (Budesonide and Formoterol), 10 atopic steroid naïve patients with MA/PR were studied before and after 12 weeks of treatment (twice daily 160 mcg/4.5 mcg). The study was approved by the Ethics Committee of the Policlinic hospital of Palermo University and complied with the Helsinki Declaration. Written informed consent was obtained from the parents of the patients enrolled in the study.

### Atopy assessment

All subjects included in the study were assessed for the atopic status by clinical history and confirmed by skin prick testing (SPT) (Stallergenes, France) performed by the use of standard prick method as previously described [11]. House dust mite (*Dermatophagoides pteronyssinus and Dermatophagoides farinae*) monosensitized patients were included in the study to select a population as much homogeneous as possible and to avoid a bias caused by other additional allergen exposure. Control group had negative SPT and no asthma or rhinitis symptoms.

### Sputum induction and processing

Sputum induction and processing was performed according to the methods of the plugs as previously described [12].

### Nasal Wash fluid collection and processing

NW fluid collection was performed by the Nebuliser connected with a disposable metered-dose nasal inhaler (Rinowash Markos – Mefar S.p.A. Bovezzo, BS, Italy). The device consisted of a plastic cup with a central compartment filled with normal saline solution (0.9% wt/vol NaCl) at 37°C, and an external compartment collecting the fluid. Total input of saline solution was 6 ml (3 ml for each nostril). The subjects were instructed to actively breathe during a Valsalva maneuver to harvest NW in the cup. Obtained samples were transferred into conical polypropylene tubes and processed as previously described by Pizzichini et al. [13] with minor modifications [14]. Briefly, dithiothreitol (DTT) (Sputolysin, Calbiochem Corp., San Diego, CA, USA), freshly prepared in a 10% dilution with distilled water, was added to the recovered NW fluid in the equivalent volume of 1/10^th^. After homogenization and centrifugation at 500 g for 10 min at ∼4°C, supernatant was stored at −80°C for later assay and cell pellet was used to prepare two cytospin slides for total cell counts. Cytocentrifuged slides were stained with May-Grunwald-Giemsa stain (Sigma Chemical Co., St. Louis. MO, USA) and examined by light microscope. The percentage of the recovered NW *versus* introduced volume was 58.3%±18.6 (mean ± SD).

### Blood sample collection and PBMC culture and stimulation

Blood samples from patients were collected in EDTA vacutainer tubes (Becton Dickinson, Mountain View, CA, USA) and used for plasma selection and PBMC isolation. The cells were isolated by density gradient centrifugation using gradient intensity (Ficoll-paque^TM^ PLUS; Amersham Biosciences SE-751 84, Uppsala, Sweden) and, after two washes, the cells were suspended in RPMI 1640 cell culture medium (Invitrogen Life Technologies, Italy) supplemented with 10% heat-inactivated FBS, 2 mM L-glutamine, 20 mM HEPES, 100 U/ml penicillin, 100 µg/ml streptomycin, 5×10^–5^ M 2-ME and 85 µg/ml gentamicin. Purity and viability were tested using trypan blue exclusion. The cells (1×10^6^ cells/ml) were cultured for 72 hours in 24-well cell culture plates in complete medium in presence or absence of PMA (50 ng/ml) (Sigma Aldrich, Italy) and ionomycin calcium salt (250 ng/ml) (Sigma Aldrich, Italy). After the selection of the dose, Budesonide 10^−8^ M and Formoterol 10^−8^ M (Italchimici S.p.A.-Italy) combination, were evaluated in the experimental conditions. The cell viability was evaluated by trypan blu exclusion at the end of each experiments, to exclude the toxicity of the drugs. The cells recovered from 10 atopic steroid naïve patients with MA/PR, studied before and after 12 weeks of treatment, were analyzed after the PBMC isolation. The cells were processed for intracellular cytokine expression and signal transduction as described forward.

### Intracellular staining of IL-17A cytokine

For the detection of intracellular IL-17A cytokine, PBMC were cultured overnight in the presence of Golgi Stop (2 μM final concentration) (Becton Dickinson, Mountain View, CA, USA). The cells were harvested and put into polypropylene tubes and then stained with anti-CD3 PE-Cy5 (Becton Dickinson, Mountain View, CA, USA) alone or with anti-CD4 FITC (Becton Dickinson, Mountain View, CA, USA) in incubation buffer (PBS containing 1% FBS and 0.1% Na azide) for 30 min at 4°C. Cells were then washed twice in PBS with 1% FBS and fixed with PBS containing 4% paraformaldehyde for 20 min at room temperature. Fixed cells were washed twice in permeabilization buffer (PBS containing 1% FBS, 0.3% saponin, and 0.1% Na azide) for 15 min at 4°C, and stained with PE anti-human IL-17A (Becton Dickinson, Mountain View, CA, USA). After two washings in PBS containing 1% FBS, the cells were analyzed by FACSCalibur supported with CellQuest acquisition and data analysis software (Becton Dickinson, Mountain View, CA, USA). Lymphocytes were gated by forward and side scatter and analysis was done on 100,000 acquired events for each sample.

### Intracellular staining of FoxP3 and RORγ(t)

PBMC were harvested and washed twice in PBS (containing 1% FBS and 0.02% Na azide). Suspended cells were stained with FITC-, PE- or PE-Cy5-conjugated antibodies against CD3, CD4 and CD25 (Becton Dickinson, Mountain View, CA, USA). Cells were fixed and permeabilized using the human FoxP3 Buffer Set (Becton Dickinson, Mountain View, CA, USA) following the manufacturer's recommended assay procedures. Finally, the cells were stained with FITC anti-human FoxP3 clone 259D/C7 (Becton Dickinson, Mountain View, CA, USA). For RORγ(t) intracellular staining, suspended cells were stained with anti-CD3 PE-Cy5 (Becton Dickinson, Mountain View, CA, USA), and fixed and permeabilized as described for FoxP3 intracellular staining. Finally cells were stained with PE anti-mouse/human RORγ(t) clone AFKJS-9 (eBioscience Inc, San Diego, CA, USA).

Cells (10^5^) from each sample were analyzed using a FACSCalibur as described above. Lymphocytes were gated by forward and side scatter and analysis was done on 100,000 acquired events for each sample.

### Epithelial cell line culture

16-HBE (a differentiated SV-40 transformed bronchial epithelial cell) and human RPMI-2650 (squamous cell carcinoma of the nasal septum) (American Type Culture Collection, ATCC, Manassas, VA, USA) cell lines represent a valid *in vitro* model to evaluate the functional properties of bronchial and nasal epithelial cells in inflammation and repair processes [15], [16]. Cell lines were grown in uncoated vented tissue culture flasks (Sarstedt, Numbrecht, Germany) and incubated at 37°C in a humidified 5% CO_2_ atmosphere. Cells were maintained in MEM (Gibco) supplemented with 10% foetal bovine serum (FBS) (Hyclone), 2mM glutamine (Euroclone), Hepes (Euroclone), gentamicin 250 μg/ml (Gibco) and 1% MEM non essential aminoacids (Gibco). The RPMI-2650 cell line was also supplemented with sodium piruvate (Lonza, Walkersville, MD, USA).

### Stimulation of epithelial cells with Ss and NW

The epithelial cells were grown as mentioned above. After 24 hrs, 16-HBE cells were coincubated with Ss from MA/PR, while RPMI-2650 cells were coincubated with NW from MA/PR, in the presence or absence of Formoterol (10^−8^ M) and Budesonide (10^−8^ M) alone or in combination, or in the presence or absence of anti IL-17R monoclonal antibody (4 μg/ml, R&D Systems, Minneapolis, MN, USA) to neutralize human IL-17R mediated bioactivity. We selected the anti-IL-17R antibody for its high ability to neutralize the biological activity of IL-17A. After 24 hrs of stimulation, cell supernatants were carefully aspirated, collected and centrifuged to remove any cell. The cell-free media were stored at −20°C for IL-8 measurements.

### Epithelial cells stimulation with exogenous IL-17A

The 16-HBE (1.0×10^5^ cells/ml) or RPMI-2650 (1.5×10^5^ cells/ml) cells were seeded in 1 mL of culture medium in 12-well cell culture plates (Falcon, Beckton Dickinson) and grown in MEM 10% FBS until 80–90% of confluence. The medium was removed and replaced with MEM supplemented with 1% FBS. After 24 hrs, the cells were stimulated in the presence or absence of rhIL-17A (50 ng/ml, R&D Systems, Minneapolis, MN, USA), in the presence or absence of Formoterol (10^−8^ M) and Budesonide (10^−8^ M) alone or in combination, or in the presence or absence of anti IL-17R monoclonal antibody (4 μg/ml, R&D Systems, Minneapolis, MN, USA) to neutralize human IL-17R mediated bioactivity.

### Measurements of IL-17A and IL-8 by ELISA

The levels of IL-17A were measured in Ss, NW and plasma from children with MA and IA, and in HC, using a commercial available enzyme-linked immunosorbent assay (ELISA) kit (R&D Systems. Inc, MN, USA). The lower limit of detection was 15 pg/ml. IL-8 release was determined in supernatants from cultured RPMI2650 and 16-HBE cells using a commercial ELISA kit (Invitrogen Corporation 542 Flynn Rd, Camarillo, CA), according to the manufactures' specifications. The lower detection limit was <5 pg/ml.

### Statistical Analysis

Statistical analysis was performed using Kruskal Wallis test and the differences between groups were evaluated by nonparametric Mann Whitney test. The results of *in vitro* experiments were analyzed using ANOVA with Fisher test correction for multiple comparisons. Statistical analysis of the data obtained in the samples before and after treatment *in vivo* was performed using the Wilcoxon test. Data were expressed as mean ± standard deviation (S.D.) A *p* value <0.05 was considered statistically significant.

## Results

### Demographic characteristics of the patients and cell count from Ss and NW samples

We reported the demographic characteristics of all patients included in the study in Table 1. The total and differential cell count of sputum and NW are reported in Table 2.

**Table 1:** Demographic characteristics of patients.

	Control	Asthma (GINA)		Overall p value*
		Intermittent	Mild – Moderate	
ARIA° Classes, (n)				
Intermittent rhinitis (Mild, Moderate/Severe)	16,00	8,00	9,00	
Persistent rhinitis (Mild, Moderate/Severe)		7,00	10,00	
Age, (years)	12.1 (±1.7)	13.9 (±3.3)	13.2 (±4.0)	ns
Range	8–15	6–17	6–17	
Sex, n (Male/Female)	16 (9/7)	15 (8/7)	19 (11/8)	ns
Body mass index, (kg/m^2^)	12.7 (±1.1)	14.0 (±2.3)	14.4 (±2.1)	ns
Range	11.2–14.8	10.6–20.3	10.6–18.7	
FEV1 (% predicted)	110.0 (±9.0)	96.3 (±11.0)	86.5 (±14.8)	<0.0001
FVC (% predicted)	95.5 (±19)	96.0 (±10.7)	89.2 (±10.4)	<0.0001
FEV1/FVC (ratio %)	89.0 (±7.0)	86.2 (±7.6)	82.1 (±8.6)	ns
PEF (% predicted)	80.6 (±14.6)	79.7 (±18.6)	60.1 (±15.7)	<0.0001
SPT, any positive, n (%)	0	100	100	<0.0001
Total IgE IU/ml, (Geometric mean)	71,94	299,80	632,72	<0.0001
log tot IgE	1.86 (±0.20)	2.48 (±0.37)	2.80 (±0.29)	<0.0001

**Table 2:** Total and differential cell count from induced sputum and nasal lavage.

	Controls	IA	MA
Induced sputum cells			
Total cell counts (10^6^ cells/ml)	0.8 (±0.7)	1.5 (±0.8)	1.2 (±0.8)
Squamous cells (%)	3.2 (±2.1)	2.7 (±2.1)	2 (±0.2)
Macrophages (%)	91 (±11.2)	56.5 (±22.3)	52.5 (±23.1)
Eosinophils (%)	0.3 (±0.2)	5.8 (±2.1)	4 (±3.8)
Neutrophils (%)	9 (±5.3)	33.5 (±2.3)	40 (±16.8)
Lymphocytes (%)	0 (±0.2)	3.5 (±0.2)	2.5 (±2.5)
Epithelial cells (%)	0 (±1.6)	0.5 (±0.3)	1 (±1.1)
Nasal lavage cells			
Total cell counts (10^6^ cells/ml)	0.01 (±0.1)	0.5 (±5.3)	0.7 (±0.2)
Macrophages (%)	17.1 (±14.3)	15.1 (±3.3)	14.3 (±5.3)
Eosinophils (%)	13.3 (±3.3)	22.1 (±5.3)	33.3 (±7.1)
Neutrophils (%)	23.8 (±15.9)	32 (±6.3)	32 (±6.3)
Epithelial cells (%)	34.4 (±32.3)	16.4 (±12.3)	8.8 (±12.3)

### Increased levels of IL-17A in allergic children

**Figure 1:**
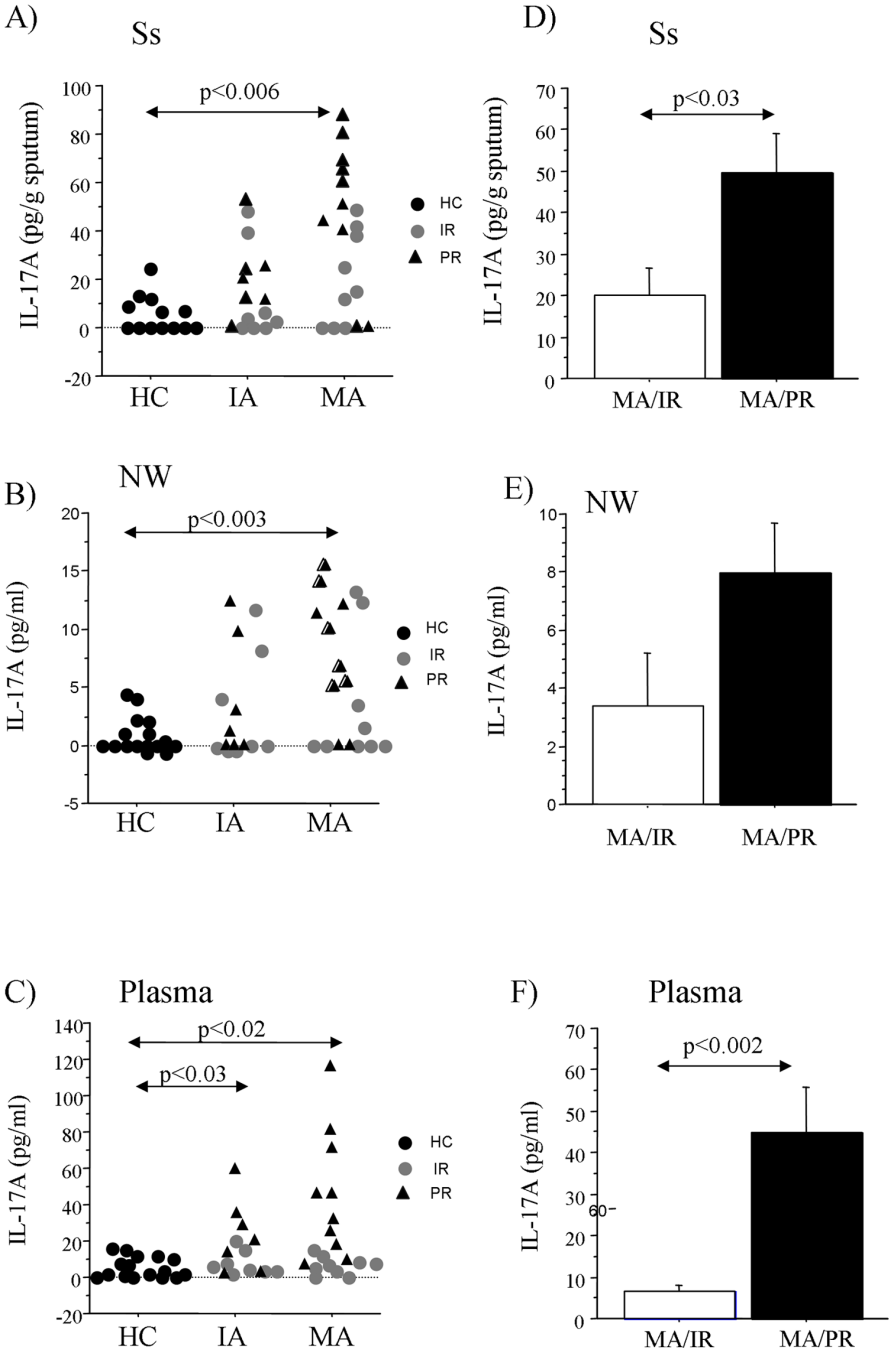
Levels of IL-17A in Control subjects (HC) and patients with asthma and rhinitis. A, B, C) IL-17A levels in Ss, NW and plasma from HC, IA, and MA groups. Results are expressed as pg/g sputum for Ss, and as pg/ml for NW and plasma. Data are shown as individual values. Statistical analysis was performed by Kruskal Wallis and Mann-Whitney tests. D, E, F) IL-17A levels in Ss, NW and plasma from MA/IR in comparison with children with MA/PR. Bars show mean ± S.D. Statistical analysis was performed by Mann-Whitney test. Significance was accepted at p<0.05.

All the subjects included in the study underwent for plasma, sputum and nasal wash samples except for three HC that did not produced sputum. IL-17A levels were significantly increased in Ss from children with MA when compared to HC (Figure 1A), and in NW from children with MA compared with HC (Figure 1B). The IL-17A plasma levels were significantly increased in children with MA and IA compared with HC. No significant differences were observed between MA and IA (Figure 1C). Furthermore, we found that IL-17A levels were significantly increased in Ss and plasma from MA/PR when compared to MA/IA (Figure 1 D, F). IL-17A levels in NW were not significantly increased in MA/PR compared to MA/IR.

### Intracellular IL-17A, RORγ(t) and FOXP3 expression in T-cells

**Figure 2:**
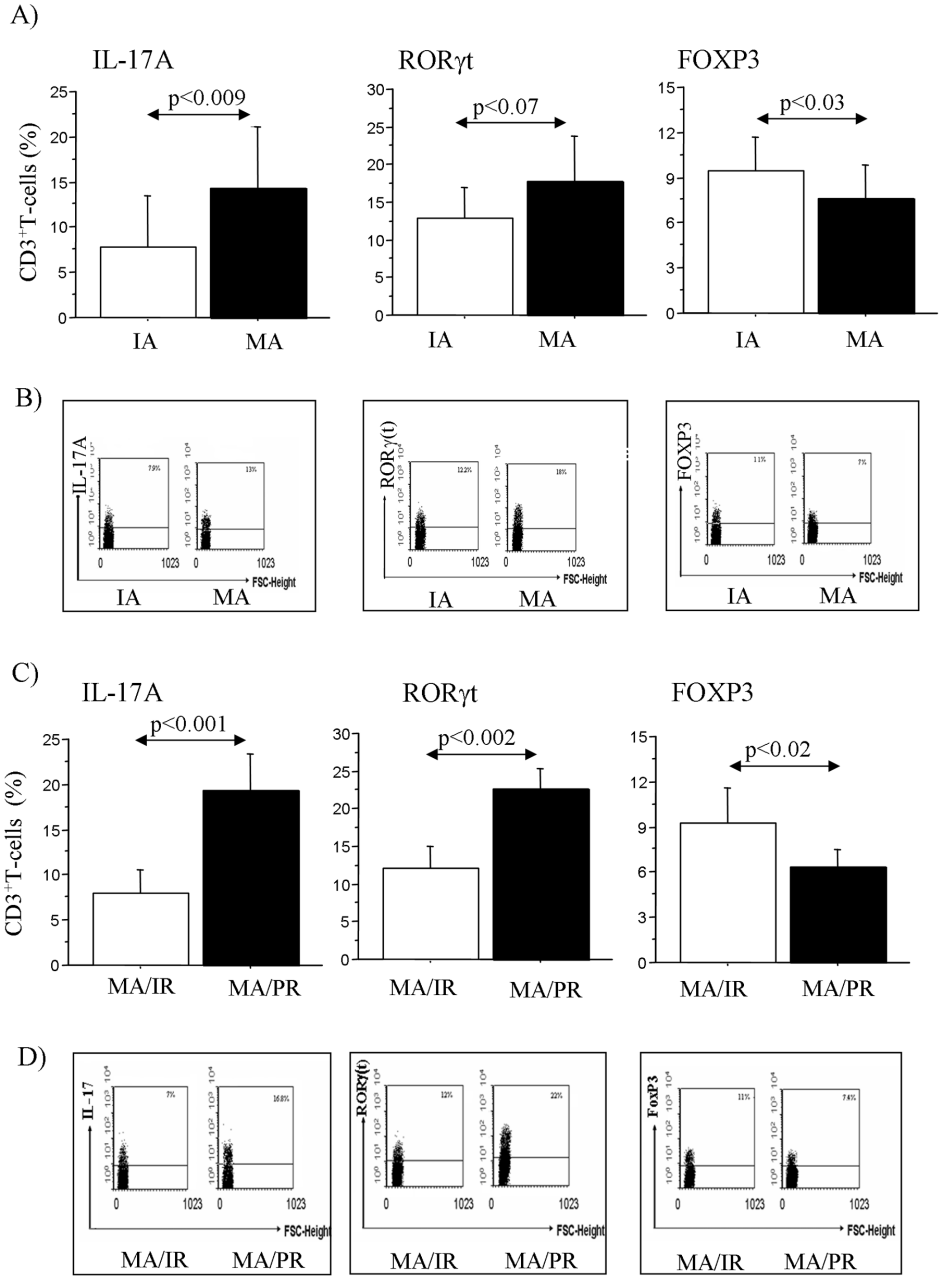
T-lymphocytes expressing intracellular IL-17A, RORγ(t) and Foxp3 in allergic children. A) Expression of CD3^+^IL-17A^+^, CD3^+^RORγ(t)^+^ and CD3^+^Foxp3^+^ in T-cells from children with IA (n = 15) and MA (n = 19); B) a representative flow cytometry of the data; C) Expression of CD3^+^IL-17A^+^, CD3^+^RORγ(t)^+^ and CD3^+^Foxp3^+^ in T-cells from children with MA/IR (n = 9) in comparison with MA/PR (n = 10); D) a representative flow cytometry of the data. T-cells (1×10^6^ cells/ml) were cultured for 72 hours in 24-well cell culture plates in complete medium in presence of PMA (50 ng/ml) and ionomycin calcium salt (250 ng/ml). The analysis was performed by flow cytometry. Gates were set on CD3^+^cells. The bars represent mean+SD of the % of CD3^+^ expressing cells. Statistical analysis was performed by Mann-Whitney test. Significance was at p <0.05.

We evaluated the percentage of T-cells expressing the intracellular markers IL-17A, RORγ(t) and FOXP3 in asthmatic patients since we found increased plasma IL-17A in MA and IA. We found a significant increase of intracellular CD3^+^IL-17A^+^ together with a statistically lower levels of CD3^+^FOXP3^+^ in MA when compared to IA, furthermore we observed higher levels of intracellular CD3^+^ RORγ(t)^+^ in this children although without a statistical significant differences (Figure 2 A, B). Finally, we observed increased intracellular levels of IL-17A and RORγ(t), and lower intracellular levels of FOXP3 in T-cells from children with MA/PR when compared to MA/IR (Figure 2C, D).

### Effect of the Budesonide and Formoterol on T-cells

**Figure 3:**
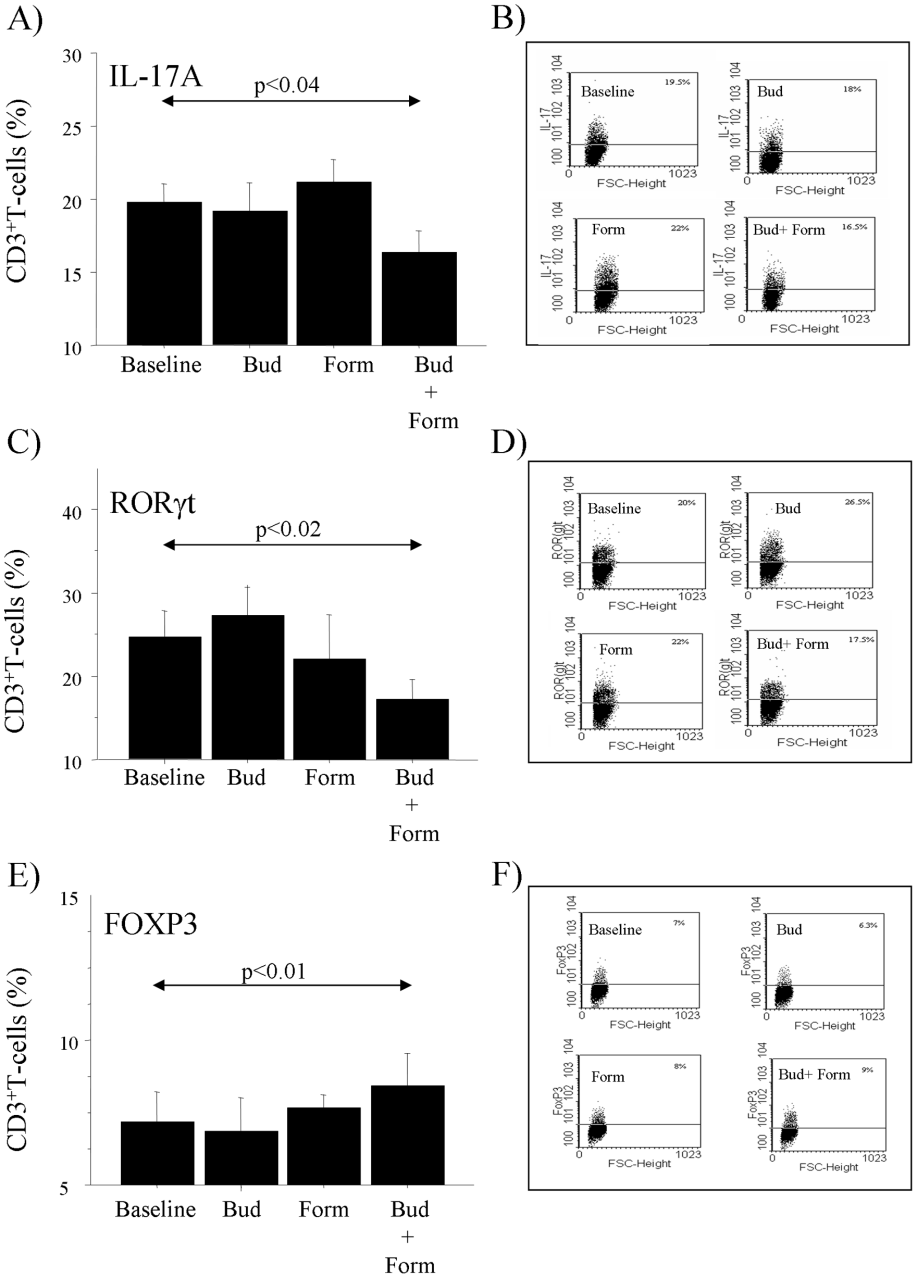
Effects of budesonide and formoterol on T-lymphocytes from children with MA/PR (n = 10). A) Expression of CD3^+^IL-17A^+^; C) Expression of CD3^+^RORγ(t)^+^; E) Expression of CD3^+^Foxp3^+^;B, D, F) representative flow cytometry of the data. The cells (1×10^6^ cells/ml) were cultured for 72 hours in 24-well cell culture plates in complete medium in presence of PMA (50 ng/ml) and ionomycin calcium salt (250 ng/ml), and then the effect of Budesonide 10^−8^ M and Formoterol 10^−8^ M alone or in combination was evaluated. The analysis was performed by flow cytometry. Gates were set on CD3^+^cells. The bars represent mean+SD of the % of CD3^+^ expressing cells. Statistical analysis was performed by ANOVA with Fisher test correction. Significance was at p <0.05.

**Figure 4:**
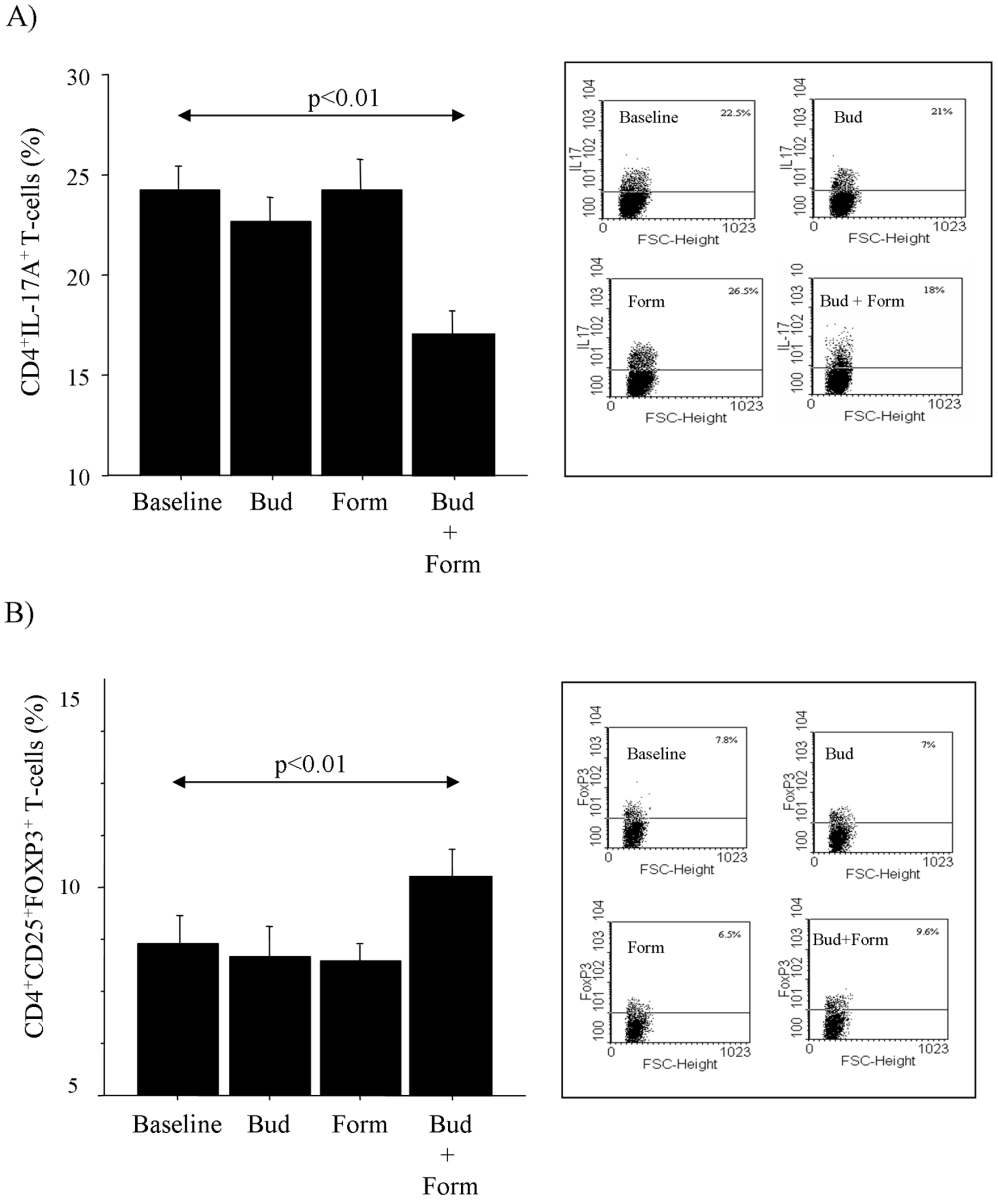
Effect of budesonide and formoterol on T-lymphocytes from children with MA/PR (n = 10). CD4^+^IL-17A^+^ and CD4^+^CD25^+^Foxp3^+^ T-cells in children with MA/PR (n = 10). A) Expression of CD4^+^IL-17A^+^; B) a representative flow cytometric detection of CD4^+^ T-cell cytokine expression in PBMC. C) Expression of CD4^+^CD25^+^Foxp3^+^; D) a representative flow cytometry analysis of CD4^+^CD25^+^ T-cells. PBMC (1×10^6^ cells/ml) were cultured for 72 hours in 24-well cell culture plates in complete medium in presence of PMA (50 ng/ml) and ionomycin calcium salt (250 ng/ml), in the presence or absence of Budesonide 10^−8^ M and Formoterol 10^−8^ M alone or in combination. The analysis was performed by flow cytometry. Gates were set on CD3^+^cells followed by CD4^+^cells and CD4^+^CD25^+^cells. Statistical analysis was performed by ANOVA with Fisher test correction. Significance was at p<0.05.

Budesonide and Formoterol alone did not affect the intracellular levels of IL-17A, RORγ(t), and FoxP3 expression in cultured T-cells from children with MA/PR when compared to baseline. Interestingly, the combined use of both Budesonide and Formoterol significantly reduced the intracellular levels of IL-17A and RORγ(t) and increased the intracellular levels of Foxp3 in T-cells from MA/PR when compared to baseline (Figure 3). Finally, since in asthma the Foxp3 expression is specifically associated with CD4^+^CD25^+^ T-cells, we investigated the Foxp3 expression in T-cells with this specific phenotype. Interestingly, the combined use of both Budesonide with Formoterol significantly reduced the intracellular levels of IL-17A in CD4^+^T-cells, while increased intracellular FOXP3 in CD4^+^CD25^+^T-cells, when compared to baseline (Figure 4).

### Effect of Budesonide and Formoterol on bronchial and nasal epithelial cells

**Figure 5:**
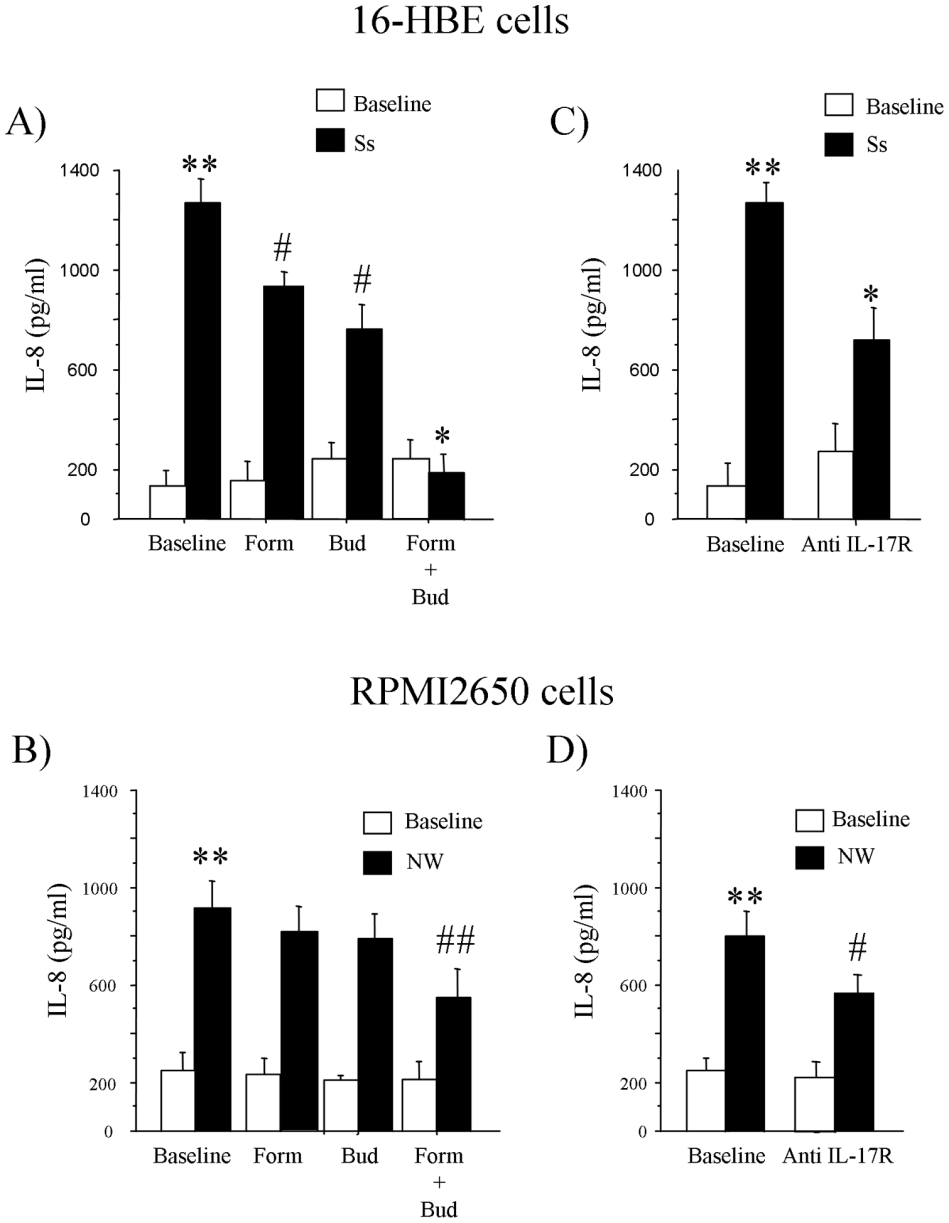
Ss and NW from children with MA/PR (n = 6) induced the IL-8 release from epithelial cells. 16-HBE and RPMI2650 cells were stimulated in the presence or absence of Budesonide 10^−8^ M and Formoterol 10^−8^ M alone or in combination A) with Ss (n = 6) or B) with NW from children with MA/PR; 16-HBE and RPMI2650 cells were stimulated in the presence or absence of anti IL-17R monoclonal antibody C) with Ss or D) with NW from children with MA/PR. Results are expressed as pg/ml. Data are shown as mean ± S.D. ANOVA with Fisher test correction was used to compare the different experimental conditions. **p<0.001 vs baseline; *p<0.001 vs IS; #*p<0.05 vs* IS. **p<0.001 vs baseline; ##*p<0.01 vs* NW; #*p<0.05 vs* NW.

**Figure 6:**
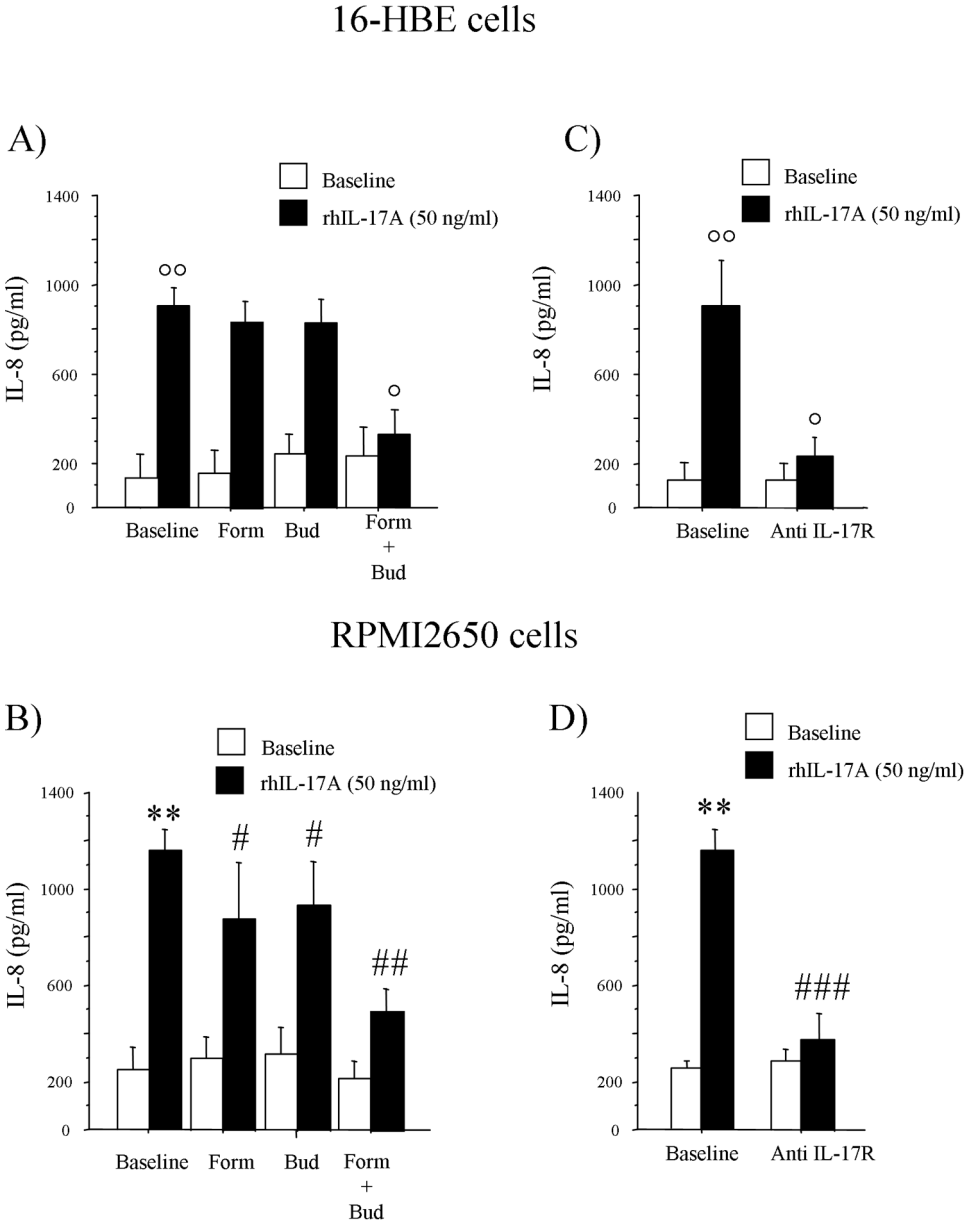
rhIL-17A induced IL-8 release from epithelial cells. 16HBE cells were stimulated with A) rhIL-17A in the presence or absence of Budesonide 10^−8^ M and Formoterol 10^−8^ M alone or in combination and B) with rhIL-17A in the presence or absence of anti IL-17R monoclonal antibody. RPMI2650 cells were stimulated with C) rhIL-17A in the presence or absence of Budesonide 10^−8^ M and Formoterol 10^−8^ M alone or in combination, and D) with rhIL-17A in the presence or absence of anti IL-17R monoclonal antibody. Results are expressed as pg/ml. Data are shown as mean ± S.D. of six separate experiments. ANOVA with Fisher test correction was used to compare the different experimental conditions. °°*p<0.001 vs* baseline; °*p<0.01 vs* rhIL-17A. ###*p<0.001 vs* rhIL-17A.

We next investigated the effect of IL-17A present in the Ss and NW from children with MA/PR on bronchial (16-HBE) and nasal (RPMI2650) epithelial cells activation. 16-HBE and RPMI2650 cells stimulated with Ss and NW from children with MA/PR significantly increased the release of IL-8 when compared to baseline. The preincubation of both bronchial and nasal epithelial cells with Budesonide or Formoterol before the stimulation with Ss and NW significantly reduced the IL-8 release; the combined drugs had a stronger synergistic effect on the reduction of IL-8 release by 16-HBE and RPMI2650 cells (Figure 5A, B). Furthermore, the neutralization of IL-17R by a specific antibody significantly reduced the Ss and NW-mediated IL-8 release from both bronchial and nasal epithelial cells (Figure 5C, D). The stimulation of 16-HBE and RPMI2650 cells with rhIL-17A showed increased levels of IL-8 release; the preincubation of the cells with Budesonide or Formoterol reduced the IL-8 production, while the combined use of both drugs had a stronger additive effect reducing the release of IL-8 mediated by rhIL-17A (Figure 6 A, B). Finally, the pretreatment of 16-HBE and RPMI2650 cells with the anti IL-17R monoclonal antibody significantly reduced the release of IL-8 generated by rhIL-17A stimulation (Figure 6 C, D).

### Effect of 12 weeks treatment with inhaled Budesonide and Formoterol

**Figure 7:**
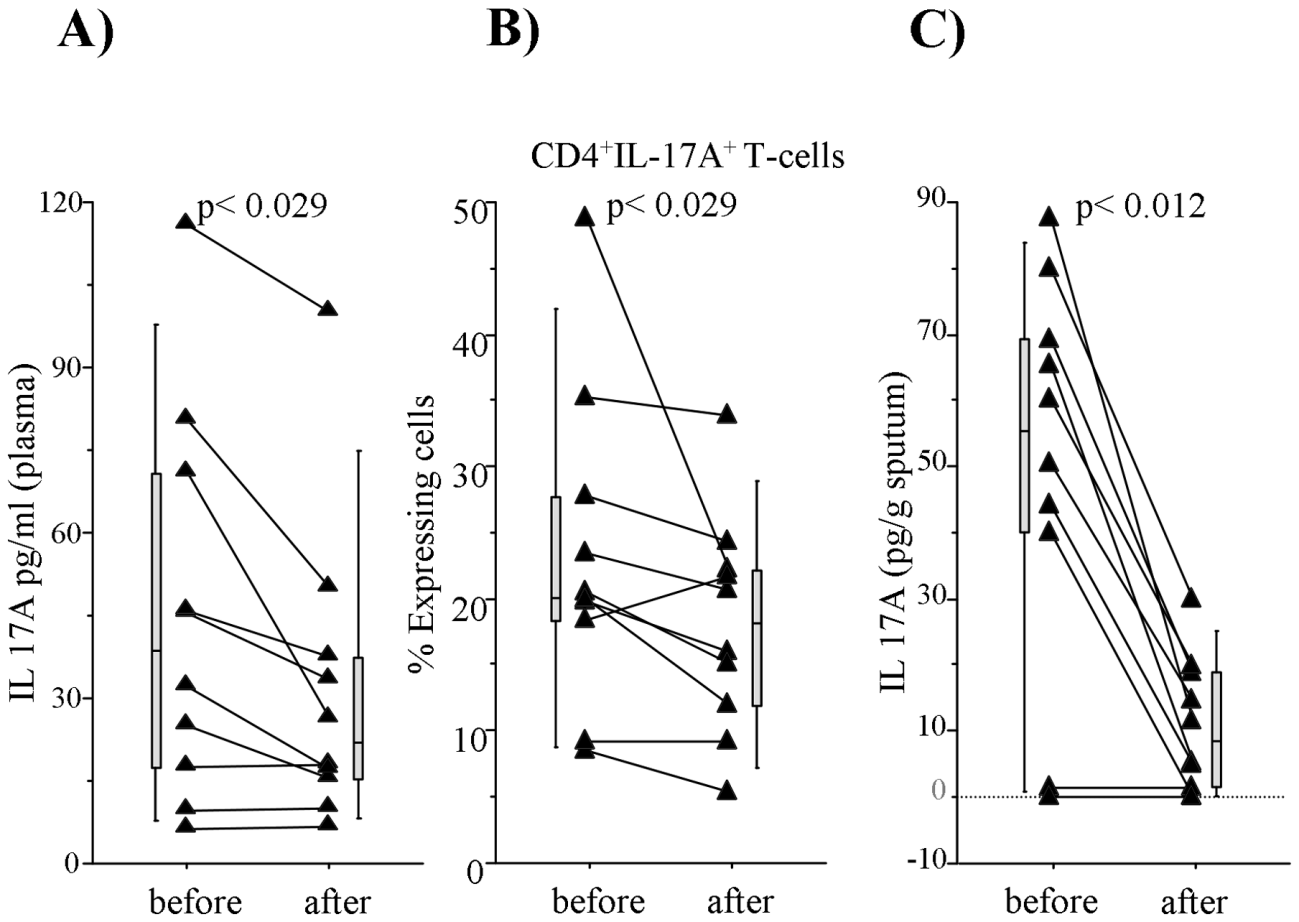
Effect of 12 weeks treatment with inhaled Budesonide and Formoterol. Pediatric patients with MA/PR were treated with Budesonide and Formoterol for 12 weeks and the effect was evaluated before and after the treatment on A) plasma IL-17A levels (pg/ml); B) CD4^+^ IL-17A^+^T-cells (%); C) Ss IL-17A levels (pg/g sputum). Results are expressed as individual data points and the lateral bars represent median (25–75 percentiles). Statistical analysis was performed by Wilcoxon U-test. Significance was at p<0.05.

To confirm *in vivo* the effects of the two drugs on the IL-17A release shown in the previous *ex vivo* and *in vitro* experiments, we tested the plasma and Ss levels of IL-17A, the percentage of CD4^+^IL-17A^+^ T-cells, in 10 patients with MA/PR at baseline and after a 12 weeks treatment with inhaled Budesonide and Formoterol, as described in Materials and Methods. As expected, the plasma levels of IL-17A (Figure 7A), the % of CD4^+^IL-17A^+^ T-cells (Figure 7B), and the Ss levels of IL-17A (Figure 7C) were significantly reduced after the 12 weeks treatment in comparison to the baseline. Finally, the % of CD4+CD25+FOXP3+T-cells showed and increased after 12 weeks of treatment in comaparison to, although without a statistical differences (data not shown).

## Discussion

**Figure 8:**
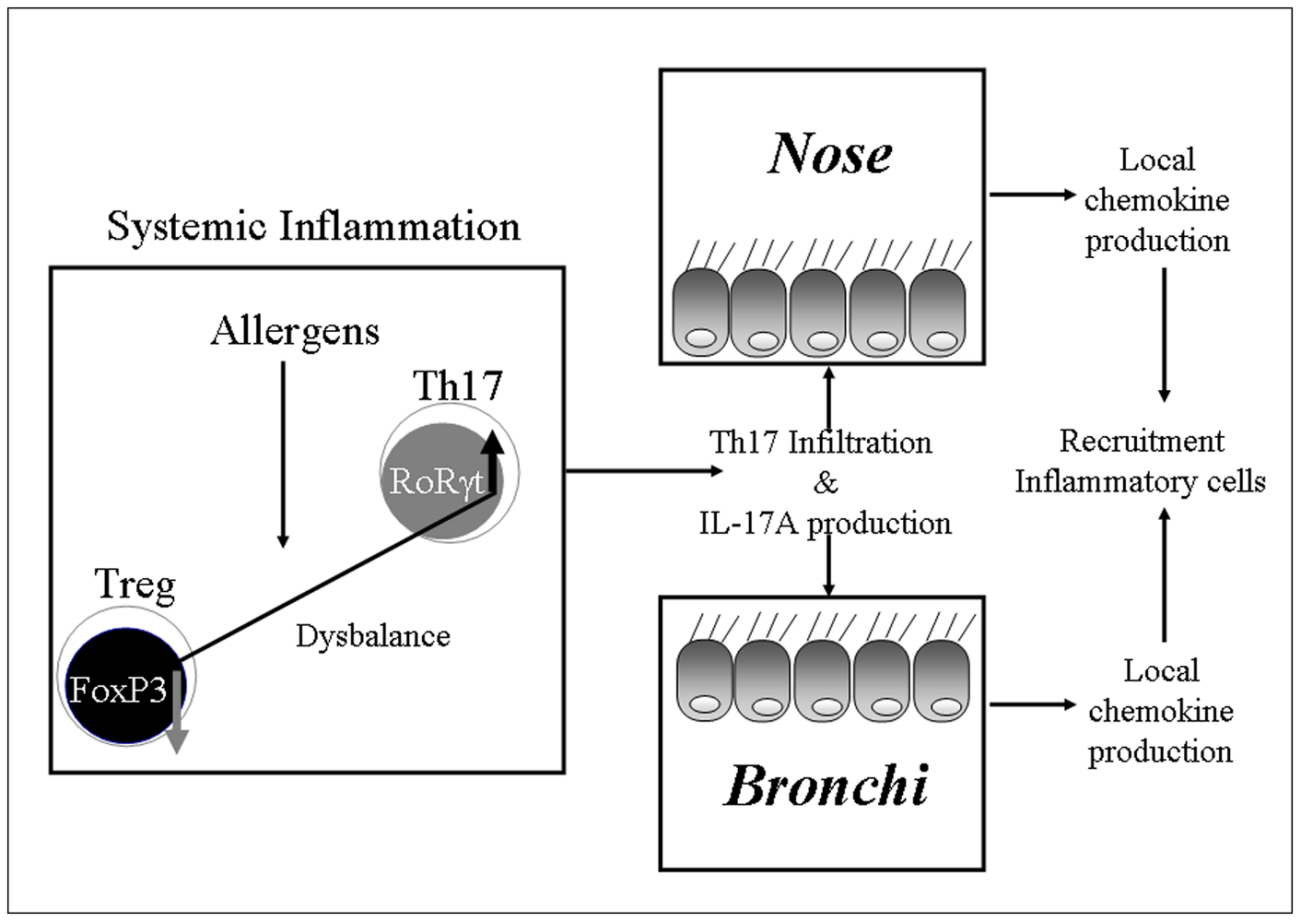
Scheme of the role of Th17/Treg dysbalance in children with asthma and rhinitis.

This study shows for the first time that increased levels of IL-17A are involved in the systemic and upper and lower airway inflammation in children with allergic asthma and rhinitis. Furthermore, showing higher levels of intracellular IL-17A and RORγ(t), associated with lower levels of Foxp3 in T-lymphocytes, together with higher plasma levels of IL-17A we suggests that there is a Th17/Treg imbalance toward Th17 cells in moderate forms of allergic asthma and rhinitis. In particular we underline that in these patients the Th17 immunity, responsible of the local inflammation in the nose and bronchi by the higher levels of IL-17A present in NW and Ss, is able to increased the production of IL-8 in nasal and bronchial epithelial cells (Figure 8). Finally this study provides informations on ICS and LABA as useful therapeutic strategies to control the Th17 immunity and IL-17A activity in children with moderate forms of asthma and rhinitis.

In asthma, airway inflammation is characterized by activation of T helper type-2 (Th-2) T cells, IgE production and eosinophilia. Uncommitted (naive) CD4^+^ T helper cells can be induced to differentiate in specific lineages of T helper type 1 (Th1), Th2, Th17 and regulatory T cell (Treg) phenotypes in a mutually exclusive manner. The phenotype of the Treg is defined as being positive for CD3, CD4, CD25 and also FoxP3, a transcription factor which is closely related to the suppressive function of Treg [17], [18]. An imbalance between Th1 and Th2 cells occurs in asthma with an increase of Th2 and a decrease of Th1 cells often due to a decrease in amount or function of Treg cells [19]. Pathogens or inflammatory mediators may impair the suppressive function of Tregs in pediatric asthma [20]. Furthermore a number of groups have recently described the conversion of Tregs into the Th17 phenotype by the activation of the nuclear receptor retinoic acid – related orphan receptor γt (RORγt) and induced by appropriate inflammatory stimuli [17], [18]. The Th17 cells produce IL-17A, a cytokine that induces the production of chemokines and antimicrobial peptides by tissue cells, leading to the recruitment of neutrophils and inflammation [4].

We demonstrate here that IL-17A is involved in systemic inflammation of children with MA rather than in children with IA and HC, accordingly to the increased levels of Th17 and RORγ(t) expression in peripheral T-lymphocytes. Furthermore, when we considered the MA group, we observed that children with PR showed higher levels of IL-17A in plasma, higher Th17 and RORγ(t) expression in peripheral T lymphocytes than children with IR. These findings suggest a role of Th17 immunity and the related released cytokines in children with the moderate forms of asthma and rhinitis. Finally, the analysis of FOXP3^+^ T-cells showed lower levels in children with MA than children with IA: in particular among MA lower levels of FOXP3^+^ T-cells were observed in children with MA/PR than children with MA/IR. All together these results support the concept that, since circulating Th17 significantly increased and Treg originally decreased in children with MA and PR, the Th17/Treg balance is impaired in these patients and may potentially play a role in the pathogenetic mechanisms as well as in the progression of allergic diseases.

IL-17 cytokines promote tissue inflammation via the induction of other pro-inflammatory cytokines and chemokines and, in humans, several studies have demonstrated that Th17 immunity is involved in the pathogenesis of allergic diseases [21] with a potential role in the severity of the disease [22], [23]. We show here higher levels of IL-17A in Ss from children with MA/PR than in children with MA/IR, contributing to further underline a potential role of IL-17A in the local airway inflammation of allergic children, and suggesting that the Th17 mediated immunity is stronger involved in children with more severe form of allergic disease. Additionally, since together with differences of Ss we did not find differences between the levels of IL-17A in NW of children with MA/PR and MA/IR, our findings support the concept that the higher levels of IL-17A in the bronchial airways, in the presence of comorbidity, might be cause of the progression of asthma disease. On the other hand, IL-17A is a proinflammatory cytokine playing a very important role in the induction and propagation of inflammation in asthma [24].

The activity of IL-17A is mediated by IL-17 receptor (IL-17R) expressed by both blood cells and structural cells including T-cells and the airway epithelial cells [25]. In this scenario, IL-17A in allergic rhinitis and asthma may cover both the innate and the adaptive aspects representing the crucial crosstalk between immune system and structural cells such as airway epithelial cells and fibroblasts [26], [27]. Several *in vitro* studies have shown that recombinant IL-17A is able to induce IL-6, IL-8, granulocyte colony-stimulating factor (GCS-F), nitric oxide (NO) and prostaglandin E2 (PGE2) in airway epithelial cells [6]. Accordingly, in our *in vitro* model we clearly identified that the stimulation with rhIL-17A promotes the release of IL-8 in both nasal and bronchial epithelial cells suggesting that IL-17A might cause the IL-8 mediated airway inflammation in the allergic disease of the nose and of the bronchi. This observation is further supported by the IL-8 released from RPMI2650 and 16HBE cells stimulated respectively with NW and Ss from children with MA/PR that was abrogated by the preincubation with an anti-IL-17AR MoAb. Finally, we observed that Ss or NW can induce a stronger release of IL-8 than rhIL-17A (ng/ml), although the levels of IL-17A in the Ss are much lower than hrIL-17A used in the experimental conditions. The explanation might be associated with the presence of proinflammatory agents in the Ss or NW that alone or synergistically with IL-17A are involved in the production of IL-8 in airway epithelial cells.

It has been demonstrated that Th17 cells producing IL-17A are able to induce neutrophilic airway inflammation in mice and that this inflammation is GC insensitive [28]. Inhaled corticosteroids (ICS) are the mainstay of anti-inflammatory treatment for asthma, often in combination with long-acting β2-agonists. The treatment with inhaled corticosteroids for controlling allergic rhinitis and asthma has proven the effectiveness in chronic persistent stages of airway diseases [29] and is consistent with systemic and airway inflammation common in all different stages of allergic disease [30]. The treatment with a combination of ICS and LABA is better than the use of higher doses of ICS alone [30] and is appropriate to counteract the mechanism of IL-17A mediated inflammation. We show here that the combined use of ICS and LABA is efficient in interfering positively with the Th17-IL-17A mediated inflammation in children, however their combined use in children remains controversial [31]. To further support the combined use of Budesonide and Formoterol we show that the two drugs are able to control the cultured Th17 phenotype by reducing the levels of intracellular IL-17A and RORγ(t), and conversely increases the levels of intracellular Foxp3 in T-lymphocytes from children with MA/PR, affecting the subtype CD4^+^IL-17A^+^ and CD4^+^CD25^+^FoxP3^+^ T-cells. These findings suggest that ICS and LABA might be able to control the Th17/RORγ(t) pathway, and can restore the Th17/Treg balance increasing the Treg in allergic children. Furthermore, our findings support the concept that using the drugs in combination the ICS are able to increase β2receptor expression in the cells, increasing the antinflammatory activity of LABA toward IL-17 activity.

Recent *in vitro* studies have demonstrated that IL-17A induces epigenetic changes which in turn diminish the ability for GCs to inhibit IL-8 production from human bronchial epithelial cells 16HBE [7]. Accordingly, the presence of bronchial inflammation associated with IL-8 was observed in the Ss from children with asthma, with higher levels in the airways of children with MA despite the ICS treatment [32]. Therefore, our in vitro results show that ICS and LABA might be able to improve the pharmacological response to the GC in the regulation of inflammatory events, generated by IL-17A present in the NW and in the Ss from children with MA/PR, and associated with IL-8 release from nasal and bronchial epithelial cell lines. Furthermore, we tested the effectiveness of ICS and LABA in a small number of patients with MA/PR treated for twelve weeks with inhaled Budesonide and Formoterol. Our finding clearly show that the association of the two drugs is able to reduce the levels of IL-17A in plasma and in the Ss, and to reduce the number of the circulating CD4^+^IL17A^+^ T-cells. All together these findings suggest that, although the combined use of ICS and LABA remains controversial in allergic disease of children, the treatment with ICS and LABA might be a useful tool for the management of systemic and local inflammation in childhood allergic disease of the airways. Further studies are necessary to better investigate the effectiveness of ICS and LABA on additional inflammatory pathways, including the levels of IL-8 produced within the airways, in children with asthma and rhinitis.

In conclusion, we have demonstrated that Th17 cells producing IL-17A are more frequently observed in allergic asthma and rhinitis in children, showing a role in the systemic and local inflammation, reinforcing the concept of the “united airway disease”. In this scenario, the understanding of the IL-17A biology might be crucial in the development of novel therapeutic approaches for the resolution of inflammation associated with crosstalk between innate and adaptive immunity during the allergic process of rhinitis and asthma, often insensitive to glucocorticosteroids treatment. Our results suggest the potential therapeutic approach with ICS and LABA to control systemic and local inflammation generated by the Th17 immunity in children with allergic airways diseases.
